# Morphological Characterisation of Haemocytes in the Mealworm Beetle *Tenebrio molitor* (Coleoptera, Tenebrionidae)

**DOI:** 10.3390/insects12050423

**Published:** 2021-05-08

**Authors:** Maria Luigia Vommaro, Joachim Kurtz, Anita Giglio

**Affiliations:** 1 Department of Biology, Ecology and Earth Science, University of Calabria, 87036 Rende, Italy; marialuigia.vommaro@unical.it; 2 Institute for Evolution and Biodiversity, University of Münster, 48149 Münster, Germany; joachim.kurtz@uni-muenster.de

**Keywords:** apoptosis, autophagy, electron microscopy, exosome, haemocyte morphology, insect immunity, mitosis, vesicular trafficking

## Abstract

**Simple Summary:**

*Tenebrio molitor* is a pest of stored grain, causing considerable damage. However, its easy maintenance makes this species also interesting as a food source and as a model for physiological, immunological, ecological and evolutionary studies. We used light and transmission electron microscopy to study the morphology of circulating haemocytes. Prohaemocytes, plasmatocytes, granular cells and oenocytoids were described based on their morphological features and staining affinity. Results are a baseline for further study aimed to clarify the structure and function of haemocytes in insects.

**Abstract:**

The immunocompetence of the mealworm beetle *Tenebrio molitor* has been well investigated at molecular and physiological levels, but information on morphological and functional characteristics of its immune cells (haemocytes) is still scarce and fragmentary. This study provides an updated overview of the morphology of circulating immune cells from mealworm beetle adults, using light and transmission electron microscopy. Based on their affinities for May–Grünwald Giemsa stain, haemocytes were defined as either eosinophilic, basophilic or neutral. Ultrastructural descriptions allowed to detect four main cell types in the haemolymph: prohaemocytes, plasmatocytes, granular cells and oenocytoids. The morphological plasticity of haemocytes and the evidence of mitotic circulating cells, intermediate cell stages, as well as autophagic activities suggest haemocyte proliferation, turnover and transdifferentiation as constantly active processes in the haemolymph. Cytochemical tests revealed differences in the distribution of carbohydrates among cell types underling the great plasticity of the immune response and the direct involvement of circulating immune cells in the resource allocation. In addition, our results provide a detailed morphological description of vesicle trafficking, macro- and microautophagy, apoptotic and necrotic processes, confirming the suitability of *T. molitor* haemocytes as a model for studying evolutionarily conserved cellular mechanisms.

## 1. Introduction

Insects rely on physical barriers, such as the cuticle, as well as cellular and humoral immune responses to counteract parasites and pathogens in their natural environments [1,2]. Cellular defences involve haemocytes, which play a crucial role in the pathogen clearance by phagocytosis, nodule formation, encapsulation and cytotoxic reactions [3,4,5,6]. Haemocyte types have mostly been characterised referring to their morphological, histochemical and functional features [7] or based on monoclonal antibodies and genetic markers [8]. The most common morphological types are prohemocytes, granular cells, plasmatocytes, spherule cells and oenocytoids, occurring in species belonging to diverse insect orders [5,9,10,11,12,13]). Humoral effectors are an efficient part of the innate immune response and include production of antimicrobial peptides, activation of prophenoloxidase (proPO) and production of reactive oxygen species [11,14]. These effectors cooperate in species-specific pathways activated to lead the recognition and neutralisation of pathogens [15]. In *Drosophila melanogaster*, phagocytic plasmatocytes, PO-containing crystal cells and lamellocytes are involved in parasite encapsulation [4,16,17]. In Lepidoptera, phagocytic granulocytes, capsule-forming plasmatocytes, spherule cells and PO-containing oenocytoids have been identified [4].

Insects such as Diptera, Lepidoptera and Coleoptera are largely used as alternative models to vertebrates in physiological, ecological and toxicological studies because of the absence of ethical restriction, short lifecycle and easy maintenance under laboratory conditions [18,19]. Due to strong structural and functional similarities in the innate immune system of insects and vertebrates [20,21], studies of insect immunity could lead to a better understanding of the evolution of innate immune systems [22]. Moreover, it represent a model to test chemicals [15,23,24,25] and bioactive molecules, including antimicrobial peptides [26] for ecotoxicological and biomedical applications. 

The mealworm beetle *Tenebrio molitor* Linnaeus, 1758 (Coleoptera, Tenebrionidae) is a pest of stored grain facilities. On the other hand, mealworm larvae are used as a source of proteins [27,28] and fatty acids [29] for animal husbandry [30] and human nutrition (EU Regulation 2017/983). Moreover, they are able to ingest and biodegrade plastic products [31,32]. Given the growing interest in their use as food and feed, there have been a number of reports investigating the cellular and humoral immune effectors of *T. molitor* in relation to the wide range of pathogens that can reduce its survival and reproductive success, as reviewed [33]. Haemocyte-mediated cellular responses to biotic challenges have been extensively studied focusing on genes encoding components of the *T. molitor* immune system [34,35,36]. To date, few studies have addressed the morphological and functional variability of haemocytes in *T. molitor*. A previous analysis using phase contrast microscopy has shown three different cell types named oenocytoids, plasmatocytes and cystocytes [37]. Scanning electron microscopy, performed to investigate abdominal haemopoietic tissues in adults, has displayed three main morphologically distinct types of haemocytes, i.e., prohaemocytes, granulocytes and plasmatocytes [38]. The response of haemocytes to biotic (*Staphylococcus aureus*) and artificial (latex beads) challenges, investigated across developmental stages, has highlighted a fourth morphological type of circulating haemocytes named oenocytoids [39]. 

The aim of this study is to characterise the circulating haemocytes of *T. molitor* based on morphological and cytochemical analyses by using light and transmission electron microscopy. We focused our attention on the ultrastructure of subcellular compartments within the different populations of circulating haemocytes to update and enhance the general knowledge of their morphological variability. 

## 2. Materials and Methods 

### 2.1. Insects 

*T. molitor* specimens were obtained from a laboratory stock population maintained at the Morphofunctional Entomology Laboratory, Dept. of Biology, Ecology and Earth Science of the University of Calabria. Mealworm beetles were reared at 60% relative humidity (rh) under a natural photoperiod and room temperature (23 ± 2), with an ad libitum diet of organic wheatmeal and fruit. In this study, adults, 7–10 days after eclosion were used.

### 2.2. Cytochemical Analyses and Light Microscopy (LM)

Before haemolymph collection, beetles were anaesthetised in a cold chamber at 4 °C for three minutes. To prepare haemocytes for wet and permanent mount staining, the haemolymph (3 μL for each specimen) was collected from beetles (n = 12) by using a 29-gauge needle at the ventral level of the pro-mesothorax articulation. It was mixed with 3 μL of phosphate buffer (PBS, 10 mM pH 7.4; Merck Life Science, Milan, Italy), put on a poly-L-lysine-coated slide and processed according to the cytochemical methods indicated below. Except for the in vivo Neutral Red assay, slides were mounted with Eukitt mounting medium (Merck Life Science, Milan, Italy) and examined under a Zeiss Primo Star microscope under immersion oil at 1000× magnification. Light microscopic images of selected areas were acquired with a Redmi note pro 9 mobile phone camera connected to the ocular at a resolution of 6000 × 8000 pixels.

- May-Grünwald Giemsa staining. To characterise the basic cellular morphology, adherent haemocytes were first fixed in pure May-Grünwald stain (Merck, Darmstadt, Germany) for 3 min. Then, slides were stained with a May-Grünwald solution (1:1 in distilled water) for 5 min, followed by a Giemsa staining (Merck) (1:20 in distilled water) for 5 min. Slides were rapidly rinsed in the distilled water, mounted and observed as indicated before. The identification of morphological cell types was based on differences in size, morphology and staining affinity.
- Phenoloxidase (PO)-haemocyte activity. PO-positive haemocytes were detected by using the method previously described in Ling et al. [40]. Briefly, as the ethanol can irreversibly activate pro-PO into PO and fix haemocytes, 1 mg/mL of L-DOPA (3,4-dihydroxy-l-phenylalanine, Sigma-Aldrich, St. Louis, MO, USA) was dissolved in 35% ethanol and the solution added to adherent haemocytes. The slides were incubated for 60 min, in dark condition, at room temperature. Control was run simultaneously by adding haemocytes with 35% ethanol. Finally, slides were rinsed with distilled water, mounted and observed as indicated before.
- Periodic acid–Schiff (PAS) staining. To detect carbohydrates, haemocytes were fixed adding a solution of 2.5% glutaraldehyde and 1% paraformaldehyde in 0.1 M phosphate buffer, pH 7.4, with 1.5% sucrose. Smears were stained with a Periodic Acid-Schiff Kit according to the manufacturer’s protocol (P.A.S. acc.Hotchkiss-Mc Manus kit, Diaphat, Bergamo, Italy). Positive cellular compartments containing polysaccharides or glycogen stained violet to pink.
- Neutral red assay. A stock solution of Neutral Red (Sigma-Aldrich) was prepared by dissolving 20 mg of the powdered dye in 1 mL of dimethyl sulphoxide (DMSO; Merck Life Science, Milan, Italy). Slides with haemolymph were incubated for 1 min in a humid chamber to allow haemocyte adhesion, then 40 µL of a working solution (Neutral Red 175 mM in PBS) prepared from the stock, was added. The retention of the neutral red in acidic intracellular compartments such as lysosomes (red) was observed in vivo under LM and acquired as indicated before.

### 2.3. Transmission Electron Microscopy (TEM)

Haemocytes were fixed and embedded as previously described [41]. Briefly, the last two abdominal segments of cold anaesthetised beetles were laterally cut and sterile PBS was slowly injected pricking the neck membrane through a 29-gauge needle. The haemolymph was quickly dropped from the abdomen into a microcentrifuge tube containing fixative (2.5% glutaraldehyde and 1% paraformaldehyde in 0.1 M phosphate buffer, pH 7.4, with 1.5% sucrose). A pool of 20 μL of haemolymph was collected from five different samples and kept at 4 °C overnight. All samples were centrifuged at 1700× *g* for 5 min and the supernatant removed. The pellets were rinsed in PBS with 1.5% sucrose, post-fixed with 1% osmium tetroxide in 0.1 M PBS for 2 h at 4 °C, then rinsed in the same buffer. The dehydration of pellets in a graded acetone series was followed by embedding in epoxy resin (Merck Life Science, Milan, Italy). Ultrathin sections, cut with a PT-PC Power Tome Ultramicrotome (RMC Boeckeler, Groot-Ammers, The Netherlands, were examined with a Jeol JEM 1400 Plus electron microscope (Microscopy and Microanalysis Centre, CM2, Laboratory of Transmission Electron Microscopy—University of Calabria, Italy) at 60 kV. Measurements were taken from the digitised images using ImageJ and processed as means ± standard error.

## 3. Results

### 3.1. Morphology and Ultrastructure of Haemocytes

**Figure 1:**
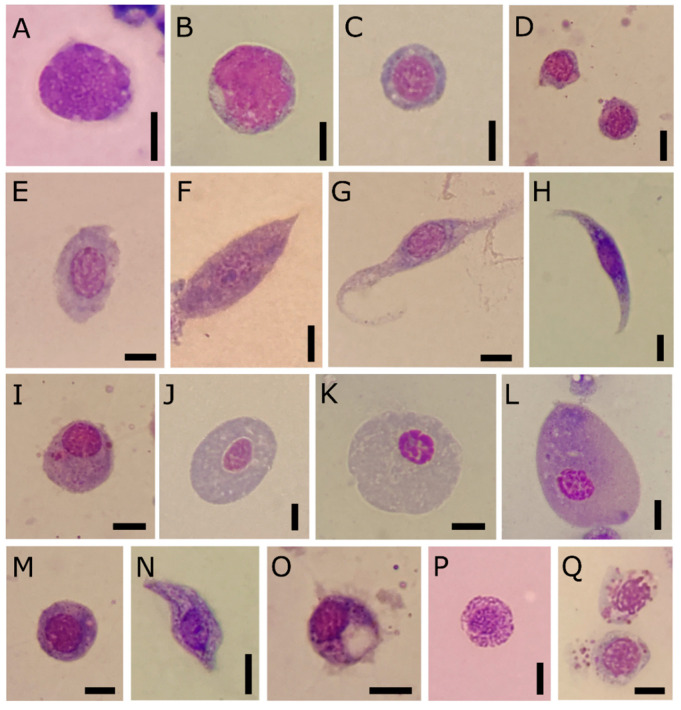
Light micrographs of haemolymph smears from *T. molitor*, May-Grünwald Giemsa stained. The metachromasia reveals specific morphological properties of different types. (**A**–**D**) Prohaemocytes. (**E**) Early plasmatocytes with large cytoplasm and heterochromatic nucleus. (**F**–**H**) Plasmatocytes as elongate cells with mature chromatin and eosinophilic cytoplasm (**F**), or spindle-shaped (**G**,**H**) with small basophilic granules in the cytoplasm. (**I**) Immature oenocytoid with eccentric nucleus and eosinophilic granules. (**J**–**L**) Differentiated oenocytoids with low nuclear-to-cytoplasmic ratio and homogenous cytoplasm. (**M**) Intermediate figure with basophilic cytoplasm and eosinophilic granules. (**N**–**O**) Circulating granular cells appear elongated in shape with large basophilic granules or round in shape and with eosinophilic granules (**P**), easily lysed and degranulated (**Q**). Bars: 5 μm.

**Figure 2:**
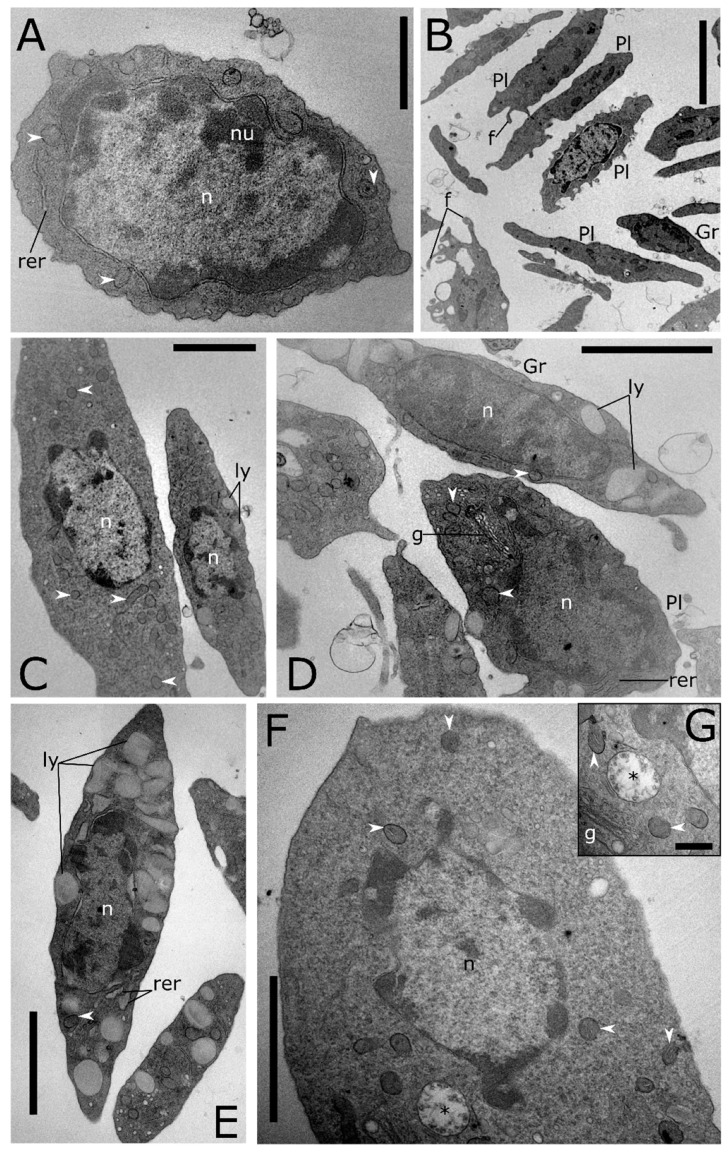
Transmission electron micrographs of haemocyte cross sections from *T. molitor*. (**A**) Prohaemocyte. (**B**) Plasmatocytes (Pl) and a granular cell (Gr). (**C**) Longitudinal section of plasmatocytes. (**D**) Granular cells (Gr) with electron-dense granules (ly) in the cytoplasm and plasmatocytes (Pl) with an evident Golgi apparatus (g). (**E**) Granular cell with granules (ly) storing homogenous electron-dense material. (**F**) Oenocytoid. (**G**) Detail of the oenocytoid showing multivesicular body (asterisk) and Golgi complex (g); n: nucleus, nu: nucleolus, rer: rough endoplasmic reticulum, white arrowheads: mitochondria. Bars: 5 µm (**B**); 2 µm (**C**–**F**); 1 µm (**A**); 500 nm (**G**).

**Figure 3:**
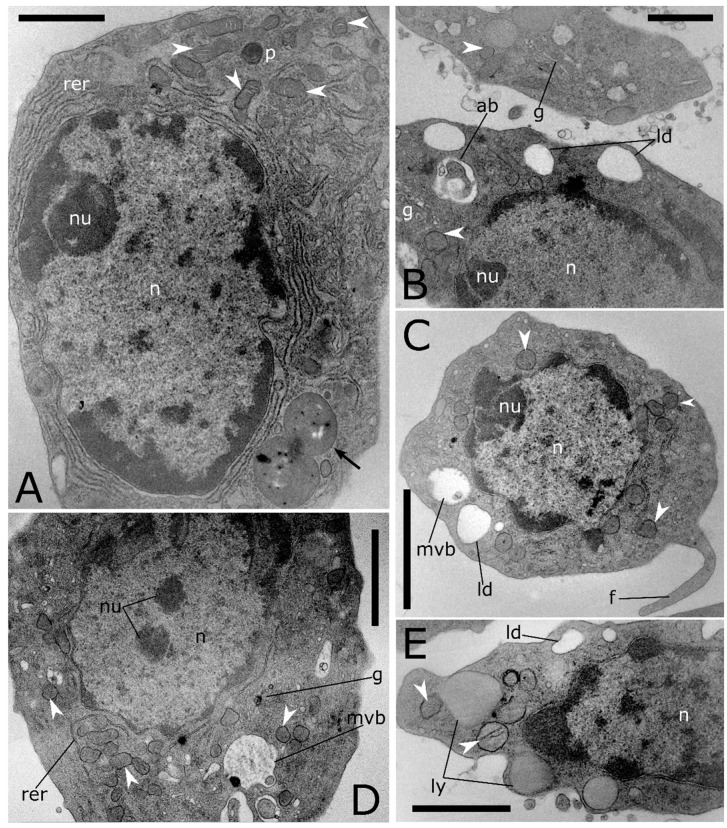
Transmission electron micrographs of haemocyte cross sections from *T. molitor*. (**A**) Intermediate haemocyte stage showing a large nucleus and a well-developed rough endoplasmic reticulum (rer). Granules with electron dense material (black arrow) are visible in the cytoplasm. (**B**) Plasmatocytes showing numerous small electron-lucent lipid droplets (ld) and an autophagic body (ab). (**C**) Transversal section of immature plasmatocytes showing an electron-lucent vesicle (ld) and a multivesicular body (mvb); plasmatic membrane is prolonged in a filopodium (f). (**D**) Intermediate cell stage without granules showing numerous mitochondria (white arrowheads), Golgi complex (g) and rough endoplasmic reticulum (rer). (**E**) Granular cell showing electron-dense granules (ly) and electron-lucent vesicles (ld). n: nucleus, nu: nucleolus, p: peroxisome. Bars: 2 µm (**D**); 1 µm (**A**–**C**); 500 nm (**E**).

**Figure 4:**
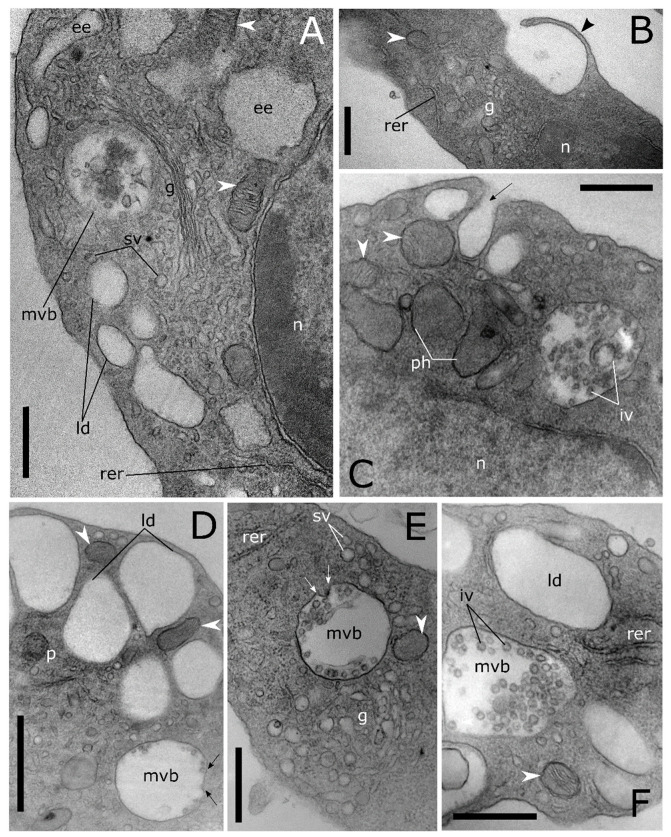
Transmission electron micrographs of cytoplasmic compartment in *T. molitor* haemocytes. (**A**) Cytoplasmic compartment showing Golgi complex (g) with secretory vesicles (sv), early endosome (ee), multivesicular body (mvb) and rough endoplasmic reticulum (rer). (**B**) Magnification of plasmatic membrane showing a phagocytic process through pseudopodium formation (black arrowhead). (**C**) Cross section showing a pinocytotic process through the plasmatic membrane invagination (black arrow). In the cytoplasm, macroautophagic processes are evident. Phagophores (ph) appear in a cup-shaped structure that engulfs a portion of the cytoplasm to form an autophagic body. (**D**–**F**) Multivesicular bodies (mvb) limiting intraluminal vesicles (iv) at the early (**D**,**E**) and late (**A**,**C**,**F**) level of the biogenesis process. Arrows (**D**,**E**) indicate intraluminal vesicles formed by budding of the limiting membrane of late endosomes. ld: lipid droplet, n: nucleus, p: peroxisome, white arrowheads: mitochondria. Bars: 1 µm (**D**); 500 nm (**A**–**C**,**E**,**F**).

**Figure 5:**
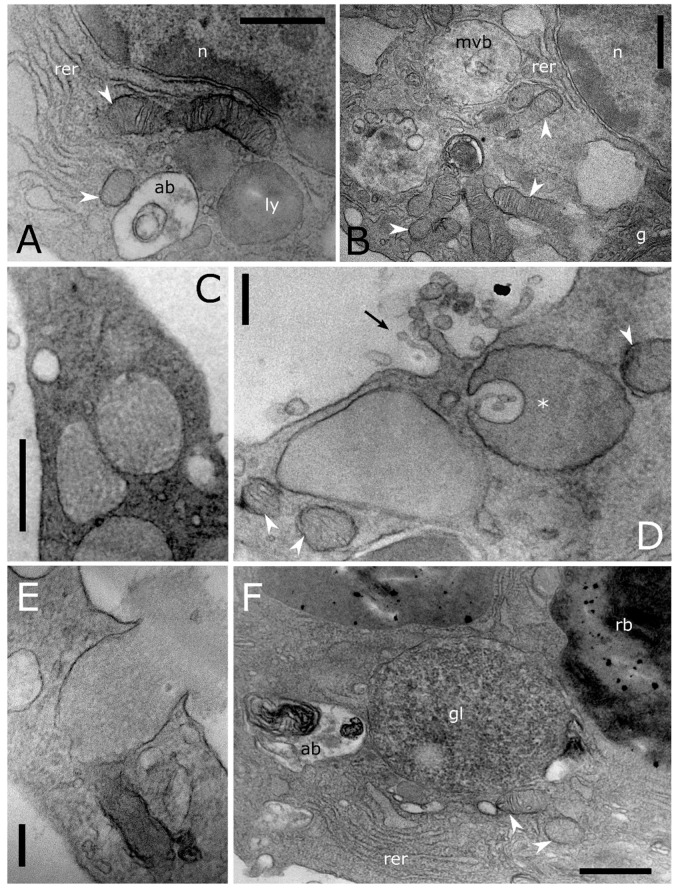
Transmission electron micrographs of cytoplasmic compartment in *T. molitor* haemocytes. (**A**,**B**) Details showing enlarged rough endoplasmic reticulum (rer), elongated mitochondria (white arrowheads) with evident cristae, autophagic body (ab), a granule (ly) storing homogeneous electron dense content and a multivesicular body (mvb). (**C**) Granules storing tubular elements. (**D**) Exosomes (black harrow) released in extracellular space by exocytic fusion of the multivesicular body with the plasma membrane. The asterisk indicates a microautophagic process. A lysosome invaginates and forms a bud sequestering cytoplasmic materials. (**E**) Degranulation process through the release of electron dense content in the extracellular medium. (**F**) Granule storing glycogen (gl) are closely adherent to a mitochondrion (white arrowhead). g: Golgi complex, n: nucleus, rb: residual body. Bars: 500 nm (**A**–**D**,**F**); 200 nm (**E**).

**Figure 6:**
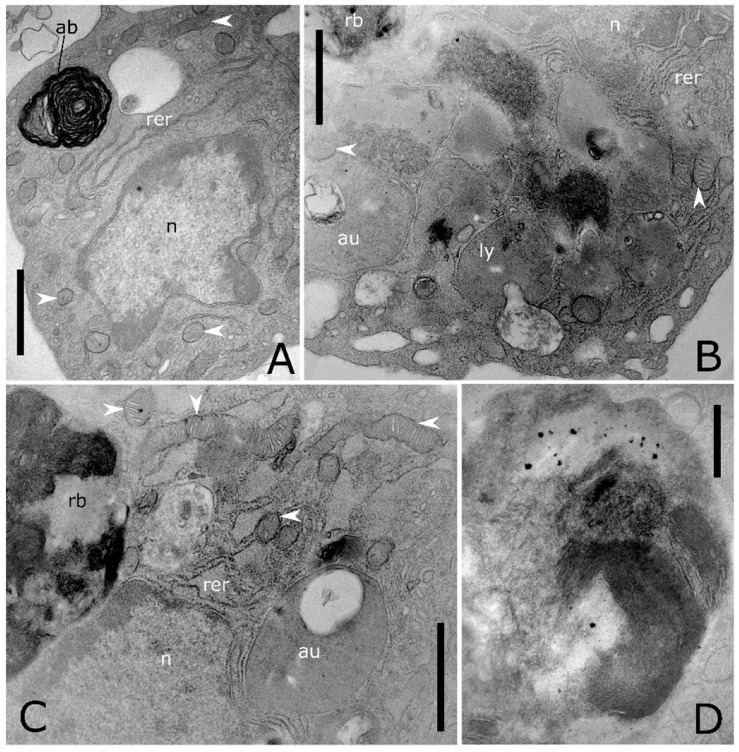
Ultrastructure of autophagic processes in *T. molitor* haemocytes. (**A**) Cytoplasm showing an autophagic body (ab) with electron dense myelinic figures inside. (**B**) Magnification of the cytoplasm showing lysosome (ly) at different stages of maturation, fusing with phagosome, featured by electron lucent appearance and storing amorphous content. (**C**) Residual body (rb) storing packed membranes and an autolysosome (au). (**D**) Detail of a residual body, electron dense content with evident myelinic figures. n: nucleus, rer: rough endoplasmic reticulum, white arrowheads: mitochondria. Bars: 1 µm (**A**–**C**); 500 nm (**D**).

**Figure 7:**
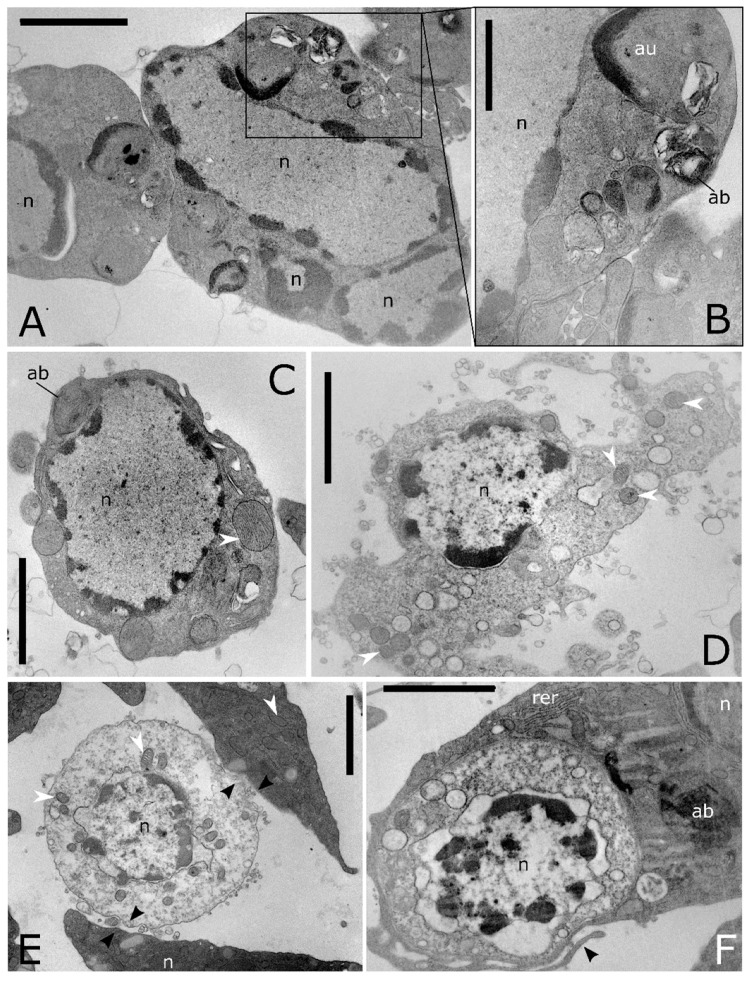
Ultrastructure of cell death in *T. molitor* haemocytes. (**A**) A cluster of apoptotic haemocytes. The chromatin condensed in patches under the inner nuclear envelope, nucleus and the plasma membrane blebbing is followed by separation of cell and nuclear fragments into apoptotic bodies. (**B**) Detail of (**A**) showing numerous macroautophagic body (ab) containing myelinic figures. (**C**) Apoptotic cell showing condensed chromatin near nuclear pores, enlarged mitochondria (white arrowhead) and macroautophagic bodies (ab). (**D**) Necrotic haemocytes with vacuolated cytoplasm, releasing cellular content in the extracellular environment. (**E**) Haemocyte at the early stage of necrosis, showing swelling of the external nuclear envelope and mitochondrial cristae, adhering trough the plasmatic membrane to other haemocytes (black arrowhead). (**F**) Cross section shows a phagocytic haemocyte wrapping a necrotic cell through prolongations of the cytoplasm (black arrowhead). au: autolysosome, n: nucleus, rer: rough endoplasmic reticulum, white arrowheads: mitochondria. Bars: 2 µm (**A**–**F**); 1 µm (**B**).

Four populations of circulating haemocyte types are identified in the haemolymph of *T. molitor* comparing their staining properties and morphology under light microscopy after May-Grünwald Giemsa staining (Figure 1A–Q), and ultrastructural analyses (Figure 2, Figure 3, Figure 4, Figure 5, Figure 6 and Figure 7). 

Prohaemocytes are immature and blast-like-cells in the haemolymph (Figure 1A–D). They appear variable in size, from 5 to 8 µm in diameter, depending on the maturation degree, and spherical in shape. The nucleus/cytoplasm ratio is 0.8 in section. The immature chromatin of the central eosinophilic nucleus is coloured from pink to violet (after May-Grünwald Giemsa staining). A limited layer of neutral cytoplasm without granules surrounds the nucleus. TEM analyses show a well-defined euchromatic nucleus, from 4 to 6 µm in diameter, and one or more prominent nucleoli, a homogeneous cytoplasm filled with a very low number of organelles such as mitochondria and rough endoplasmic reticulum (RER) (Figure 2A).

Plasmatocytes appear as elongate or spindle-shaped cells from 9 to 15 µm in length and from 3 to 5 µm in diameter in the ultimate stage of differentiation (Figure 1F–H). Their cytoplasm is neutral to basophilic (blue after May-Grünwald Giemsa staining) with small granules. Ultrathin sections of plasmatocytes show the lobate euchromatic nucleus (2.6 × 3.3 µm in diameter) with a well-developed nucleolus (Figure 2B–D). Numerous organelles such as a well-developed RER, Golgi complexes and numerous elongate mitochondria (up to 1 µm) occur in the cytoplasm (Figure 2B,D). A low number of electron lucent vacuoles 1 µm in diameter are also present (Figure 3B,C). The plasmatic membrane extends a variable number of filopodia (Figure 2B).

Oenocytoids are compared to the other cell types encountered in the haemolymph. They are elliptical cells characterised by an eccentric nucleus. The nucleus/cytoplasm ratio is approximatively 0.4 in section (Figure 1J–L). The cytoplasm appears homogeneous and neutral or slightly basophilic (blue) in LM. The ultrastructural analyses show cells, approximately 5 × 15 µm in diameter, with few organelles in the cytoplasm such as small mitochondria, free ribosomes, rough endoplasmic reticulum, Golgi complex and some multivesicular bodies (Figure 2F,G). The nucleus (4 µm in diameter) is small and heterochromatic.

Granular cells are usually elongated, with an acidophilic nucleus (red after May-Grünwald Giemsa method staining) (Figure 1N–Q) and the cytoplasm slightly basophilic (blue). Ultrathin sections show cells from 8 to 11 in length and from 3 to 6 in width (Figure 2D,E). The main distinctive feature is the presence in the cytoplasm of large granules, which are electron dense round vesicles with a diameter from 0.4 µm to 0.9 µm, storing homogenous or structured tubular elements (Figure 2D,E). 

A high level of cell-to-cell variability is found within these four populations of haemocytes. Intermediate cell stages sharing features related to different types are commonly recorded at LM (Figure 1E,I,M) and TEM analyses (Figure 3). The variability concerning the cytoplasmic compartment suggests cellular maturation throughout different functional stages (Figure 3A–E). In some cases, the rough endoplasmic reticulum (RER) is well-developed and filled the cytoplasm indicating an active protein biosynthesis (Figure 3A,D). Filopodia appear as finger-like processes at the plasmatic membrane level (Figure 3C). Lipid droplets closely associated with mitochondria and endoplasmic reticulum (Figure 3B,C) are found in the population of haemocytes identified as plasmatocytes. Electron-dense granules closely associated with mitochondria are evident in the cytoplasm of haemocytes identified as granular cells (Figure 3E). A number of vesicles are evident in the cytoplasm (Figure 4A–E) close to the trans cisterna of the Golgi complex (Figure 4A) likely related to the cellular vesicular trafficking, which involves endo (Figure 4B,C) and exocytic (Figure 5E) pathways, lysosomes and early and late endosomes including multivesicular bodies (MVBs) (Figure 4A,C–F). MVBs (approximatively 1 μm in diameter) were clearly identifiable adjacent to the plasma membrane trapping numerous intraluminal vesicles (44 ± 1 nm in diameter, n = 35) (Figure 4A,B,E,F). The mechanism for packing of bioactive molecules into exosomes, through vesicles budding and pinching off into the MVBs lumen, appears evident at different developmental levels in the cytoplasm (Figure 4D–F). MVBs are located close to the Golgi complex at the crossroads between the biosynthetic and endocytic routes of haemocytes (Figure 4A,E). Intraluminal vesicles are released in extracellular space by exocytic fusion of MVBs with the plasma membrane (Figure 5D). A variable number of granules occur in the cytoplasm. The primary type is an electron-dense round membrane-limited inclusion able in storing (Figure 5A,B,D) and exocyting (Figure 5E) enzymes, very abundant in the haemocytes identified as granular cells. The second type is an electron-opaque granule including densely packed microtubular elements (Figure 5C). Granules storing glycogen are also evident (Figure 5E). In the nucleus, the amount of heterochromatin is most abundant in cells that are less or not active.

**Figure 8:**
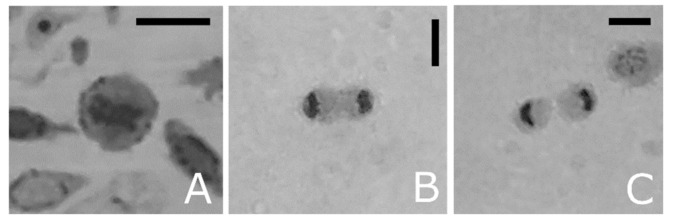
Light microscopy images of haemocyte semithin sections from *T. molitor*, showing mitotic stages. (**A**) Metaphase. (**B**) Telophase, (**C**) Cytokinesis. Bars: 5 µm.

Ultrathin sections show also haemocytes with large autophagic compartments (Figure 6A–C). The different phases of the macroautophagic pathway are evident in the ultrathin sections from phagophores enveloping organelles (Figure 4C and Figure 5A) to autophagosome (Figure 6A–D) that fuses with lysosomes (Figure 5A) forming autolysosomes (Figure 6B,C). Autolysosomes contain digested cellular material and organelles at different stages of degeneration (Figure 6B–D). Moreover, the lysosome membrane pinches off into the organelle lumen and engulfs cytoplasmic material forming microautophagic bodies (Figure 5D). Myelin-like figures, which represent the results of autophagic degradation of membranous cellular components were evident (Figure 5F and Figure 6A). Apoptotic (Figure 7A–C), degranulated (Figure 7D) and necrotic haemocytes (Figure 7E) occurring in the haemolymph are entrapped from other circulating haemocytes (Figure 7E,F). Cells occurring in various phases of mitotic division (Figure 8A–C) indicate a turnover of haemocytes.

### 3.2. Cytochemical and Cytoenzymatic Analyses

**Figure 9:**
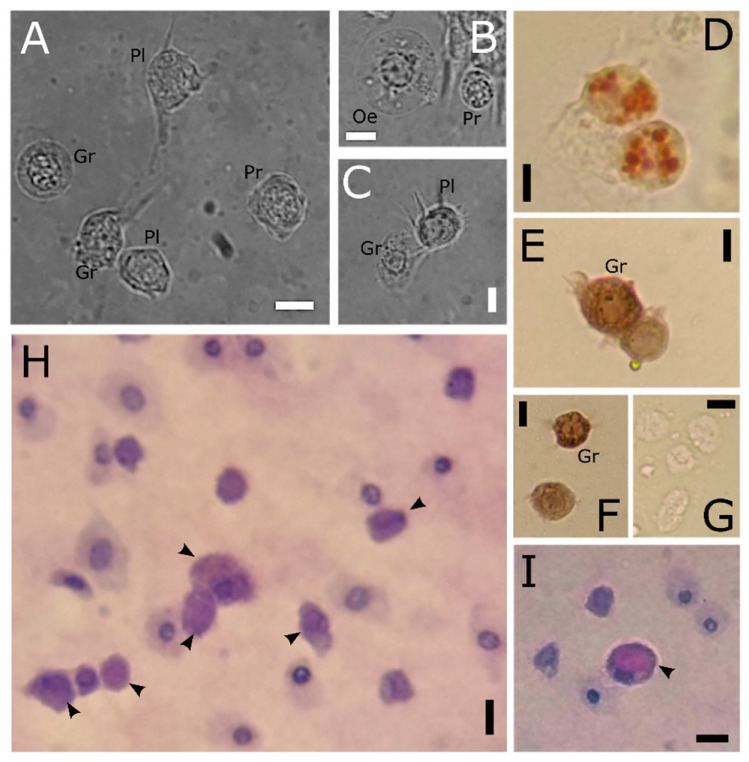
Light microscopy images of haemolymph smears from *T. molitor*. (**A**–**C**) In vivo haemocytes. (**D**) Haemocytes stained with neutral red to highlight lysosomes in red. (**E**–**G**) PO-haemocyte activity. Haemocytes showing cytoplasmic localisation of phenoloxidase in granular cells (**E**,**F**) and control (**G**). (**H**,**I**) PAS staining. Positive sites (arrowheads) indicating stored carbohydrates. Gr: granular cells; Oe: oenocytoids; Pl: plasmatocytes; Pr: prohaemocytes. Bars: 5 µm.

Haemocytes of *T. molitor* display diverse responses to cytochemical and cytoenzymatic analyses (Figure 9). Granular cells and plasmatocytes are able to accumulate the neutral red dye (Figure 9D), revealing acidic vacuolar compartments and the nature of granules as lysosomes. The circulating haemocytes are only partly positive to the PAS reaction, revealing a different distribution of carbohydrates among cell types (Figure 9H,I). PAS reaction positivity is observed in the cytoplasmic compart outside of granules. Oenocytoids (Figure 9I) are also recorded as PAS positive, while variable responses are observed for other cell types. Cytoenzymatic analysis of haemocytes reveals the presence of prophenoloxidase in almost all cell types. The strongest reaction of the zymogen activation is observed in granular cells (Figure 9E,F), granules of which were stained orange/brown. The controls did not show any reaction product (Figure 9G).

## 4. Discussion

This study provides a comprehensive description of the haemocyte ultrastructure in *T. molitor* adults. The results highlighted four morphological types of circulating haemocytes we referred to as prohaemocytes, plasmatocytes, granular cells and oenocytoids according to a previous study carried out by using fluorescence and scanning electron microscopy [39]. Actually, circulating haemocytes have been described in a low number of coleopteran species. Nevertheless, morphology and function are rather variable among the species described so far with different life histories [42,43,44]. Four to seven morphologically distinct types were identified, named prohemocytes, plasmatocytes, granular cells, coagulaocytes, oenocytoids, adipohemocytes and spherulocytes in haemolymph of Coccinellidae [45], Curculionidae [46], Melolonthidae [47], Scarabeaidae [10,48], Chrysomelidae [49] and Carabidae [12,13,41,50]. Granular cells, plasmatocytes and oenocytoids have been indicated as the main cell types involved in the phagocytosis in both adults and larvae [10,12,45,46,48]. Here, we found that granular cells are the main PO-positive circulating haemocytes of *T. molitor*, as observed in previous studies [40,51,52]. Thus we assume that they may be involved in the melanisation cascade or in aggregation processes, such as encapsulation and nodulation of pathogens [53]. Moreover, the large amount of lysosomes found in the granular cells confirmed the capability to internalise foreign organisms into a phagosome as indicated in previous studies [39,54] and a role in removal and degradation of necrotic cells and apoptotic bodies as observed in ultrathin sections. 

The haemocyte classification is still a controversial topic and limitations to perform a comparative analysis among the low number of species so far described are partly due to the differences of investigation methods besides the species-specific variability [42]. Moreover, there is an intrinsic cell-to-cell variability related to the haemocyte function that is misidentified as an indicator of morphological diversity. Previous studies hypothesised a separate immutable cell lines differentiating from haemopoietic organs or circulating prohaemocytes [4,55,56]. However, cytochemical and ultrastructural analyses performed in *T. molitor* allowed us to observe phenotypic variation within each cellular population. This variability deals with the assumption of a limited number of circulating haemocyte types involved in cellular defences, that we considered different stages with separate functions. As a result, prohaemocytes are only less differentiated cells, as observed in our ultrastructural analyses, instead of multipotent stem cells, coherently with previous studies [13,17,57,58]. Indeed, the conversion of already differentiated circulating haemocytes into another cell type has been observed in vitro in plasmatocytes of *T. molitor*, *Periplaneta americana*, *Galleria mellonella* [59], *D. melanogaster* [60] and in prohemocytes of *Bombyx mori* [61]. Previous studies showed that morphological features, such as the size of nucleoli indicate a high proteosynthetic activity closely related to the ability of a cell to differentiate into another cell type [62,63]. According to this finding, haemocytes here indicated as plasmatocytes, which show prominent nucleoli in an euchromatic nucleus and a well-developed rough endoplasmic reticulum, should be considered as one of the earliest stages of functionally differentiated cells. On the contrary, oenocytoids should have lowered potential to turn into another type. Thus, we assume that the morphological differences observed among and within the cellular populations of *T. molitor* may be closely related to the variation of the cell function during its life cycle. This hypothesis is supported by the high number of haemocytes found in both smears and ultrathin sections, showing very similar subcellular structure and micro and macroautophagic activities involved to maintaining the regular homeostatic turnover of organelles [64,65]. Moreover, the evidence of mitotic circulating cells suggests that haemocyte proliferation occurs to replace apoptotic and necrotic haemocytes and not just as a response following infection as observed in *D. melanogaster* [58]. Future studies may include techniques such as single cell RNA-sequencing to reveal hidden complexity and achieve a more fine-grained characterisation of haemocyte types related to their function as predicted from gene expression patterns. However, up to now, this technique has to our knowledge only been applied to a few insect model organisms such as *D. melanogaster* [66] and *Anopheles gambiae* [67].

The PAS positive-stained cells and ultrastructure of granules storing glycogen and lipids indicated that haemocytes of *T. molitor* may be involved in the regulation of carbohydrate and lipid levels in the haemolymph. As observed in larvae of *D. melanogaster*, lamellocytes become dependent on a massive supply of carbohydrates to perform the encapsulation of parasitoid wasp eggs [68]. Thus, autophagy also promotes cellular homeostasis by facilitating nutrient utilisation. The sequestration of polysaccharide such as glycogen in the autophagosomes and its subsequent degradation in the autolysosomes is a selective process activated under conditions of demand for massive production of glucose as observed in vertebrates [69] and insects [70]. Indeed, the plasticity of the immune response requested to face pathogens during the insect life has maintenance costs [71] and leads to a systemic metabolic switch, redirecting the metabolic resources to the activated immune system. As neuropeptides play an important immunotropic role in the regulation of the insect cellular and humoral responses promoting the mobilisation of energetic sources, such as the breakdown of glycogen into circulating carbohydrates [72,73] and lipid [74], *T. molitor* may be a useful model to study the endocrine regulation of the immune responses in insects.

The large number of MVBs found in the cytoplasmic compartment provided evidences of a cell-to-cell communication involving exosomes. MVBs are late endosomes containing intraluminal vesicles, formed through direct inward budding of their limiting membranes, that move up to the cell surface, fuse with the plasma membrane and release single-membrane exosomes outside the cell [75]. In insects, the role of exosomes in cell polarity and intracellular communication is a new emerging topic [76,77,78,79,80,81,82]. Exosomes have been isolated for the first time from the haemolymph of *Allomyrina dichotoma* (Coleoptera: Scarabaeidae) [83]. Thus, further investigation on the molecular content of these vesicles in *T. molitor* will improve knowledge about cell-to-cell communication. 

This study contributes to increasing knowledge on the function and morphology of haemocytes in *T. molitor*. The ultrastructure will be a baseline for further studies aimed to clarify the relationship between structure and function of the subcellular compartment in haemocytes and their role to transfer information through the haemocoel. Moreover, our findings validate the suitability of haemocytes in *T. molitor* to study cellular evolutionary conserved processes, such as vesicular trafficking and macro and microautophagy.

## Data Availability

All microscopy data are available in the Laboratory of Morphofunctional Entomology, Department of Biology, Ecology and Earth Science, University of Calabria, Italy.
